# Community mental healthcare in lebanon

**DOI:** 10.1192/j.eurpsy.2021.979

**Published:** 2021-08-13

**Authors:** R. Charara, J. El-Khoury

**Affiliations:** Psychiatry, American University of Beirut Medical Center, Beirut, Lebanon

**Keywords:** Eastern Mediterranean, global mental health, community mental health, psychiatric services

## Abstract

**Introduction:**

Lebanon is a medium-income country in the Eastern Mediterranean which has seen a surge in interest in mental health following years of stagnation. The mental health needs of the country for severe psychiatric disorders are underserved.

**Objectives:**

The aim of our study is to describe community mental healthcare services in Lebanon and to address local opportunities and challenges.

**Methods:**

A review of the literature using local resources along with expert opinion was undertaken to synthesize the evidence.

**Results:**

Political instability, chronic underfunding and widespread stigma have contributed to maintaining a traditional model of private clinics affiliated with inpatient and long-stay psychiatric units. A number of initiatives have been launched to cater for patients with psychotic disorders and to offer partial hospitalization for others with mood-related conditions. In parallel, the Ministry of Public Health, with international funding, has been instrumental in efforts to standardize care at a national level, particularly for early detection and treatment in primary care. The priorities of the national mental health programme are consistent with the global trend in shifting services to the community. Hurdles remain, in line with those facing countries with similar socio-demographics and resources. These include limited third-party coverage of mental health, absence of training opportunities in multidisciplinary community settings and some clinicians’ reluctance to update their ways of working.

**Conclusions:**

Development of a local workforce dedicated to providing a patient-centred approach in the least restrictive settings, is essential for consolidating community care in Lebanon. This would be reinforced by (overdue) legislation and implementation of a mental health law.

